# 

**Published:** 1886-11

**Authors:** 


CONTENTS.

Vol. 33 No. 11

| | PAGE. |
|---|---|
| Special | 298 |
| Dreams cont'd | 298 |
| Prof. Joseph Rhodes Buchanan | 302 |
| Magnetic and Electric Forces | 308 |
| Signs of the Times | 311 |
| Continental Hereditary Atonic Dyspepsia | 313 |
| Twenty Years in a Trance | 314 |
| Book Notices | 315 |
| Home Department | 316 |
| Miscellaneous Items | 318 |
| False and True | 319 |

INDEX TO ADVERTISEMENTS OF HALF A PAGE AND OVER.

| | PAGE. |
|---|---|
| Fellows' Hypo-Phosphites | I |
| Lactated Food | II |
| Celerina | III |
| Lactopeptine | IV |
| Castoria | IV |
| Horsford's Acid Phosphate | V |
| Caulocorea | V |
| Bovinine | VI |
| Vaginal Injecting and Suction Syringe | VIII |
| Offer Extraordinary | IX |
| Medicated Suppositories | X |
| Dr. Buckland's Scotch Oats Essence | 2d page cover |
| Perforated Paper | 3d " " |
| Compound Oxygen | 4th " " |

